# 6-Methyl-4-{[4-(tri­methyl­sil­yl)-1*H*-1,2,3-triazol-1-yl]meth­yl}-2*H*-chromen-2-one

**DOI:** 10.1107/S2414314620004277

**Published:** 2020-04-03

**Authors:** Sowmiya Carmel Y., N. L. Prasad, Noor Shahina Begum, Hari Prasad Suresh

**Affiliations:** a Department of Studies in Chemistry, Bangalore University, Jnana Bharathi Campus, Bangalore-560 056, Karnataka, India; b Department of Studies in Chemistry, Bengaluru Central University, Central College Campus, Bengaluru-560 001, Karnataka, India; University of Aberdeen, Scotland

**Keywords:** crystal structure, coumarin, weak C—H⋯O inter­actions

## Abstract

In the title compound, C_16_H_19_N_3_O_2_Si, the dihedral angle between the coumarin ring system (r.m.s. deviation = 0.031 Å) and the triazole ring is 73.81 (8)°. In the crystal, mol­ecules are linked into [010] chains by weak C—H⋯O inter­actions.

## Abstract 2

In the crystal of the title compound, the mol­ecules are linked into [010] chains by C—H⋯O inter­actions.

## Graphical abstract

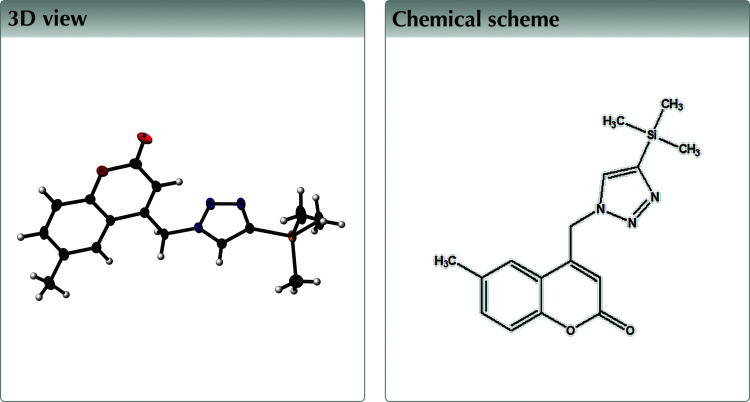

## Structure description

Coumarins are a family of benzopyrones and are widely distributed in nature (Venugopala *et al.*, 2013 ▸). They have been extensively studied as a result of their broad array of biological activities, low toxicity and low drug resistance properties (Lipeeva *et al.*, 2019 ▸). As part of our work in this area, we now describe the synthesis and crystal structure of the title compound in which the coumarin ring system bears a tri­methyl­silyl triazole substituent.

**Figure 1:**
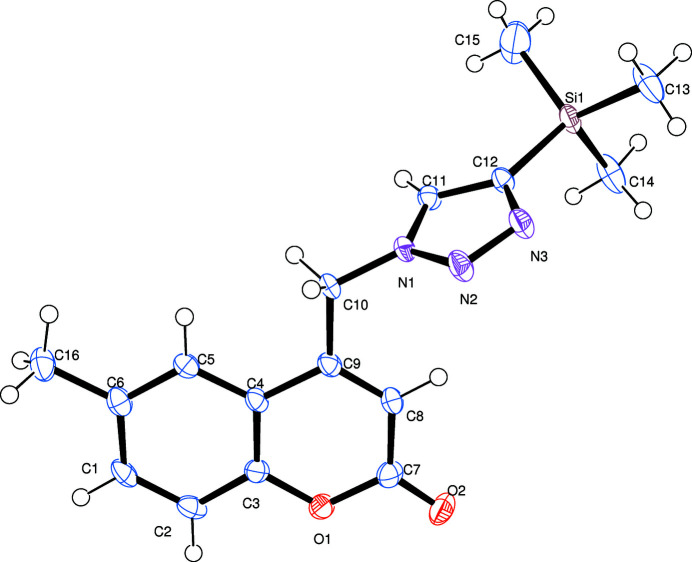
The mol­ecular structure of the title compound with displacement ellipsoids drawn at the 50% probability level. labels on the small side and displaced a long way from their respective atoms

**Figure 2:**
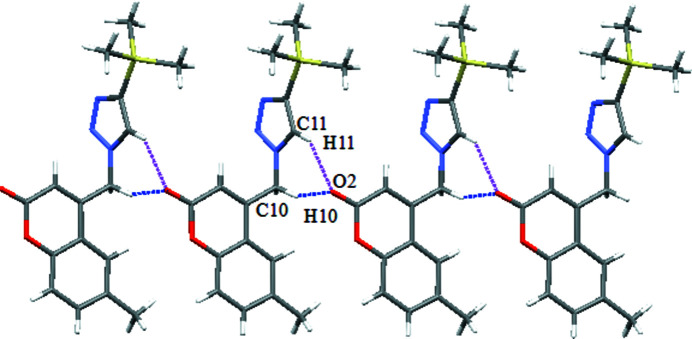
Part of a [010] chain in the crystal of the title compound showing C—H⋯O inter­actions as dotted lines. H-atoms not involved in hydrogen bonding have been excluded.

The title compound crystallizes in the monoclinic crystal system in space group *C*2/*c* with one mol­ecule in the asymmetric unit (Fig. 1 ▸). The dihedral angle between the C1–C9/O1 chromen-2-one fused ring system (r.m.s. deviation = 0.031 Å) and the N1–N3/C11/C12 1,2,3-triazole ring is 73.81 (8)°. In the crystal, weak C—H⋯O hydrogen bonds (Table 1 ▸) link the mol­ecules into [010] chains, with atom O2 accepting two such bonds from the adjacent mol­ecule (Fig. 2 ▸) related by simple translation.

## Synthesis and crystallization

Tri­methyl­silyl acetyl­ene (2.00 mmol) was added dropwise over a period of 30 min to an ice-cold suspension of bromo­methyl­coumarin (2.00 mmol), sodium azide (1.50 mmol) and copper iodide (1 µmol) in 10 ml (1:1 *v*/*v*) water/acetone. The resulting mixture was allowed to warm to room temperature and stirred for 8 h: progress of the reaction was monitored by TLC and GC through micro-workup of aliquots. After the completion of the reaction as indicated by the chromatograms, the excess acetone was removed under rotary evaporation and the crude product was purified by column chromatography, using silica gel (100–200 mesh) and 2:5 ethyl acetate–petroleum benzine (60–74°C fraction) eluent to obtain the title compound as a buff-coloured solid (91%); melting point: 110–112°C; (KBr disk, cm^−1^): 3126, 2920, 2850, 1705, 1573, 1492, 1382, 1247, 1193, 1116, 1056, 950, 825, 756, 630, 557, 509; ^1^H NMR (400 MHz, CDCl_3_): δ 0.34 (*s*, 9 H), 2.42 (*s*, 3 H), 5.74 (*s*, 2 H), 5.92 (*s*, 1H), 7.26–7.57 (*m*, 4 H, 3 H of coumarinyl aromatic protons and 1 H of triazoyl aromatic proton); ^13^C NMR (100 MHz, CDCl_3_): δ −1.0, 21.2, 49.5, 114.9, 116.9, 117.4, 123.4, 129.6, 133.8, 134.8, 148.2, 148.5, 151.9, 160.3; MS: calculated 313.12, found *m*/*z* (relative abundance) 313.22 (9.75%), 314.26 (*M* + 1 = 3.45%), 73.13 (100%); CHNS: Calculated C: 61.31%, H: 6.11%, N:13.41% Found C: 60.92%, H: 6.04%, N: 13.33%. Colourless blocks of the title compound were recrystallized from ethanol solution.

## Refinement

Crystal data, data collection and structure refinement details are summarized in Table 2 ▸.

## Supplementary Material

Crystal structure: contains datablock(s) global, I. DOI: 10.1107/S2414314620004277/hb4344sup1.cif

Structure factors: contains datablock(s) I. DOI: 10.1107/S2414314620004277/hb4344Isup2.hkl

Infrared spectrum. DOI: 10.1107/S2414314620004277/hb4344sup3.tif

1H NMR Spectrum. DOI: 10.1107/S2414314620004277/hb4344sup4.tif

13C NMR spectrum. DOI: 10.1107/S2414314620004277/hb4344sup5.tif

MS. DOI: 10.1107/S2414314620004277/hb4344sup6.tif

Supporting information file. DOI: 10.1107/S2414314620004277/hb4344Isup7.cml

CCDC reference: 1993489

Additional supporting information:  crystallographic information; 3D view; checkCIF report

## Figures and Tables

**Table 1:** Hydrogen-bond geometry (Å, °)

*D*—H⋯*A*	*D*—H	H⋯*A*	*D*⋯*A*	*D*—H⋯*A*
C10—H10*A*⋯O2^i^	0.99	2.61	3.427 (1)	140
C11—H11⋯O2^i^	0.95	2.45	3.242 (1)	141

Symmetry code: (i) 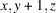 .

**Table 2:** Experimental details

Crystal data	
Chemical formula	C_16_H_19_N_3_O_2_Si
*M*_r_	313.43
Crystal system, space group	Monoclinic, *C*2/*c*
Temperature (K)	100
*a*, *b*, *c* (Å)	20.869 (2), 6.5971 (6), 24.561 (2)
β (°)	103.419 (4)
*V* (Å^3^)	3289.1 (5)
*Z*	8
Radiation type	Mo *K*α
μ (mm^−1^)	0.15
Crystal size (mm)	0.14 × 0.14 × 0.12

Data collection	
Diffractometer	Bruker SMART APEX CCD
Absorption correction	Multi-scan (*SADABS*; Bruker, 1998 ▸)
*T*_min_, *T*_max_	0.979, 0.982
No. of measured, independent and observed [*I* > 2σ(*I*)] reflections	21620, 3589, 2644
*R*_int_	0.071
(sin θ/λ)_max_ (Å^−1^)	0.639

Refinement	
*R*[*F* ^2^ > 2σ(*F* ^2^)], *wR*(*F* ^2^), *S*	0.054, 0.125, 0.99
No. of reflections	3589
No. of parameters	203
H-atom treatment	H atoms treated by a mixture of independent and constrained refinement
Δρ_max_, Δρ_min_ (e Å^−3^)	0.42, −0.31

Computer programs: *SMART* (Bruker, 1998 ▸), *SAINT-Plus* (Bruker, 1998 ▸), *SHELXS97* and *SHELXL97* (Sheldrick, 2008 ▸) and *CAMERON* (Watkin *et al.*, 1996 ▸).

## full crystallographic data

### Crystal data

**Table:**

C_16_H_19_N_3_O_2_Si	*F*(000) = 1328
*M**_r_* = 313.43	*D*_x_ = 1.266 Mg m^−^^3^
Monoclinic, *C*2/*c*	Mo *K*α radiation, λ = 0.71073 Å
*a* = 20.869 (2) Å	Cell parameters from 3589 reflections
*b* = 6.5971 (6) Å	θ = 2.9–27.0°
*c* = 24.561 (2) Å	µ = 0.15 mm^−^^1^
β = 103.419 (4)°	*T* = 100 K
*V* = 3289.1 (5) Å^3^	Block, colorless
*Z* = 8	0.14 × 0.14 × 0.12 mm

### Data collection

**Table:**

Bruker SMART APEX CCD diffractometer	3589 independent reflections
Radiation source: fine-focus sealed tube	2644 reflections with *I* > 2σ(*I*)
Graphite monochromator	*R*_int_ = 0.071
ω scans	θ_max_ = 27.0°, θ_min_ = 2.9°
Absorption correction: multi-scan (SADABS; Bruker, 1998)	*h* = −26→26
*T*_min_ = 0.979, *T*_max_ = 0.982	*k* = −8→8
21620 measured reflections	*l* = −31→31

### Refinement

**Table:**

Refinement on *F*^2^	Primary atom site location: structure-invariant direct methods
Least-squares matrix: full	Secondary atom site location: difference Fourier map
*R*[*F*^2^ > 2σ(*F*^2^)] = 0.054	Hydrogen site location: inferred from neighbouring sites
*wR*(*F*^2^) = 0.125	H atoms treated by a mixture of independent and constrained refinement
*S* = 0.99	*w* = 1/[σ^2^(*F*_o_^2^) + (0.0517*P*)^2^ + 6.1123*P*] where *P* = (*F*_o_^2^ + 2*F*_c_^2^)/3
3589 reflections	(Δ/σ)_max_ < 0.001
203 parameters	Δρ_max_ = 0.42 e Å^−^^3^
0 restraints	Δρ_min_ = −0.31 e Å^−^^3^

### Special details

**Table:**

Geometry. All s.u.'s (except the s.u. in the dihedral angle between two l.s. planes) are estimated using the full covariance matrix. The cell s.u.'s are taken into account individually in the estimation of s.u.'s in distances, angles and torsion angles; correlations between s.u.'s in cell parameters are only used when they are defined by crystal symmetry. An approximate (isotropic) treatment of cell s.u.'s is used for estimating s.u.'s involving l.s. planes.
Refinement. Refinement of F^2^ against ALL reflections. The weighted R-factor wR and goodness of fit S are based on F^2^, conventional R-factors R are based on F, with F set to zero for negative F^2^. The threshold expression of F^2^ > 2σ(F^2^) is used only for calculating R-factors(gt) etc. and is not relevant to the choice of reflections for refinement. R-factors based on F^2^ are statistically about twice as large as those based on F, and R- factors based on ALL data will be even larger.The H atoms were placed at calculated positions in the riding-model approximation with C—H = 0.95 Å, 1.00 Å and 0.96 Å for aromatic, methyne and methyl H-atoms respectively, with *U*_iso_(H) = 1.5*U*_eq_(C) for methyl H atoms and *U*_iso_(H) = 1.2*U*_eq_(C) for other hydrogen atoms.

### Fractional atomic coordinates and isotropic or equivalent isotropic displacement parameters (Å^2^)

**Table:**

	*x*	*y*	*z*	*U*_iso_*/*U*_eq_	
Si1	0.03716 (3)	0.5449 (10)	0.15123 (2)	0.02394 (18)	
O1	0.23596 (8)	−0.0948 (2)	0.43023 (6)	0.0249 (4)	
O2	0.15849 (8)	−0.2426 (2)	0.36664 (6)	0.0326 (4)	
N1	0.20048 (8)	0.3969 (3)	0.26552 (7)	0.0191 (4)	
N2	0.20697 (9)	0.2741 (3)	0.22317 (7)	0.0254 (4)	
N3	0.15636 (9)	0.3094 (3)	0.18156 (7)	0.0250 (4)	
C1	0.35583 (11)	0.2153 (4)	0.52305 (8)	0.0252 (5)	
H1	0.3807	0.2112	0.5607	0.030*	
C2	0.31413 (11)	0.0572 (3)	0.50291 (9)	0.0247 (5)	
H2	0.3101	−0.0547	0.5262	0.030*	
C3	0.27813 (10)	0.0645 (3)	0.44800 (8)	0.0202 (5)	
C4	0.28295 (10)	0.2265 (3)	0.41304 (8)	0.0187 (4)	
C5	0.32612 (10)	0.3849 (3)	0.43475 (8)	0.0210 (5)	
H5	0.3304	0.4963	0.4114	0.025*	
C6	0.36260 (10)	0.3823 (3)	0.48958 (9)	0.0234 (5)	
C7	0.19476 (11)	−0.0985 (3)	0.37792 (9)	0.0247 (5)	
C8	0.19909 (11)	0.0696 (3)	0.34119 (8)	0.0223 (5)	
H8	0.1708	0.0714	0.3048	0.027*	
C9	0.24152 (10)	0.2231 (3)	0.35664 (8)	0.0195 (4)	
C10	0.24919 (10)	0.3949 (3)	0.31845 (8)	0.0207 (5)	
H10A	0.2464	0.5245	0.3381	0.025*	
H10B	0.2936	0.3870	0.3107	0.025*	
C11	0.14617 (10)	0.5105 (3)	0.25053 (8)	0.0190 (4)	
H11	0.1309	0.6089	0.2728	0.023*	
C12	0.11697 (10)	0.4563 (3)	0.19648 (8)	0.0191 (4)	
C13	0.03782 (12)	0.5050 (5)	0.07685 (9)	0.0384 (7)	
H13A	0.0740	0.5828	0.0678	0.058*	
H13B	−0.0042	0.5505	0.0531	0.058*	
H13C	0.0440	0.3606	0.0703	0.058*	
C14	−0.03101 (12)	0.3935 (4)	0.16733 (9)	0.0368 (6)	
H14A	−0.0733	0.4511	0.1476	0.055*	
H14B	−0.0280	0.3963	0.2077	0.055*	
H14C	−0.0278	0.2531	0.1552	0.055*	
C15	0.02778 (15)	0.8163 (4)	0.16805 (14)	0.0603 (10)	
H15A	0.0610	0.8968	0.1554	0.091*	
H15B	0.0337	0.8320	0.2086	0.091*	
H15C	−0.0163	0.8320	0.1490	0.091*	
C16	0.40799 (11)	0.5535 (4)	0.51336 (9)	0.0297 (5)	
H16A	0.4063	0.6577	0.4846	0.045*	
H16B	0.4531	0.5021	0.5255	0.045*	
H16C	0.3942	0.6124	0.5454	0.045*	

### Atomic displacement parameters (Å^2^)

**Table:**

	*U*^11^	*U*^22^	*U*^33^	*U*^12^	*U*^13^	*U*^23^
Si1	0.0243 (3)	0.0291 (4)	0.0149 (3)	0.0056 (3)	−0.0026 (2)	−0.0034 (3)
O1	0.0367 (9)	0.0208 (8)	0.0168 (7)	−0.0033 (7)	0.0049 (7)	0.0003 (6)
O2	0.0487 (11)	0.0256 (9)	0.0241 (8)	−0.0140 (8)	0.0100 (7)	−0.0068 (7)
N1	0.0222 (9)	0.0219 (9)	0.0119 (8)	0.0000 (8)	0.0012 (7)	−0.0007 (7)
N2	0.0279 (10)	0.0316 (11)	0.0148 (9)	0.0071 (9)	0.0012 (7)	−0.0039 (8)
N3	0.0261 (10)	0.0320 (11)	0.0147 (9)	0.0060 (8)	−0.0001 (7)	−0.0019 (8)
C1	0.0281 (12)	0.0331 (13)	0.0122 (10)	0.0068 (10)	0.0006 (9)	0.0015 (9)
C2	0.0313 (12)	0.0252 (12)	0.0178 (10)	0.0067 (10)	0.0059 (9)	0.0058 (9)
C3	0.0250 (11)	0.0187 (11)	0.0169 (10)	0.0023 (9)	0.0051 (8)	−0.0010 (8)
C4	0.0217 (11)	0.0215 (11)	0.0125 (9)	0.0030 (9)	0.0030 (8)	−0.0011 (8)
C5	0.0225 (11)	0.0233 (11)	0.0169 (10)	0.0010 (9)	0.0041 (8)	0.0024 (8)
C6	0.0215 (11)	0.0299 (12)	0.0184 (11)	0.0022 (10)	0.0038 (9)	−0.0022 (9)
C7	0.0346 (13)	0.0217 (12)	0.0191 (11)	−0.0010 (10)	0.0087 (9)	−0.0034 (9)
C8	0.0264 (11)	0.0246 (12)	0.0142 (10)	−0.0007 (10)	0.0013 (8)	−0.0017 (9)
C9	0.0225 (11)	0.0211 (11)	0.0146 (10)	0.0035 (9)	0.0035 (8)	−0.0004 (8)
C10	0.0224 (11)	0.0235 (11)	0.0129 (10)	−0.0006 (9)	−0.0024 (8)	0.0011 (8)
C11	0.0218 (11)	0.0206 (11)	0.0148 (10)	0.0009 (9)	0.0047 (8)	−0.0004 (8)
C12	0.0206 (10)	0.0228 (11)	0.0134 (9)	0.0013 (9)	0.0029 (8)	−0.0003 (8)
C13	0.0306 (13)	0.0660 (19)	0.0164 (11)	−0.0041 (13)	0.0008 (10)	0.0070 (11)
C14	0.0266 (12)	0.0652 (19)	0.0174 (11)	0.0048 (13)	0.0028 (9)	−0.0006 (11)
C15	0.0529 (19)	0.0388 (17)	0.070 (2)	0.0193 (15)	−0.0244 (16)	−0.0181 (15)
C16	0.0290 (12)	0.0383 (14)	0.0198 (11)	−0.0060 (11)	0.0014 (9)	−0.0023 (10)

### Geometric parameters (Å, º)

**Table:**

Si1—C13	1.849 (2)	C6—C16	1.502 (3)
Si1—C14	1.854 (3)	C7—C8	1.446 (3)
Si1—C15	1.858 (3)	C8—C9	1.341 (3)
Si1—C12	1.869 (2)	C8—H8	0.9500
O1—C7	1.370 (3)	C9—C10	1.503 (3)
O1—C3	1.375 (3)	C10—H10A	0.9900
O2—C7	1.207 (3)	C10—H10B	0.9900
N1—C11	1.337 (3)	C11—C12	1.373 (3)
N1—N2	1.350 (2)	C11—H11	0.9500
N1—C10	1.453 (2)	C13—H13A	0.9800
N2—N3	1.309 (2)	C13—H13B	0.9800
N3—C12	1.375 (3)	C13—H13C	0.9800
C1—C2	1.375 (3)	C14—H14A	0.9800
C1—C6	1.401 (3)	C14—H14B	0.9800
C1—H1	0.9500	C14—H14C	0.9800
C2—C3	1.384 (3)	C15—H15A	0.9800
C2—H2	0.9500	C15—H15B	0.9800
C3—C4	1.389 (3)	C15—H15C	0.9800
C4—C5	1.402 (3)	C16—H16A	0.9800
C4—C9	1.453 (3)	C16—H16B	0.9800
C5—C6	1.385 (3)	C16—H16C	0.9800
C5—H5	0.9500		
			
C13—Si1—C14	108.43 (11)	C4—C9—C10	117.21 (18)
C13—Si1—C15	112.47 (15)	N1—C10—C9	114.30 (17)
C14—Si1—C15	110.24 (15)	N1—C10—H10A	108.7
C13—Si1—C12	109.47 (10)	C9—C10—H10A	108.7
C14—Si1—C12	109.08 (10)	N1—C10—H10B	108.7
C15—Si1—C12	107.11 (11)	C9—C10—H10B	108.7
C7—O1—C3	121.84 (16)	H10A—C10—H10B	107.6
C11—N1—N2	110.77 (16)	N1—C11—C12	106.13 (18)
C11—N1—C10	128.60 (17)	N1—C11—H11	126.9
N2—N1—C10	120.63 (17)	C12—C11—H11	126.9
N3—N2—N1	106.71 (16)	C11—C12—N3	106.42 (17)
N2—N3—C12	109.96 (16)	C11—C12—Si1	128.90 (16)
C2—C1—C6	121.8 (2)	N3—C12—Si1	124.57 (14)
C2—C1—H1	119.1	Si1—C13—H13A	109.5
C6—C1—H1	119.1	Si1—C13—H13B	109.5
C1—C2—C3	118.6 (2)	H13A—C13—H13B	109.5
C1—C2—H2	120.7	Si1—C13—H13C	109.5
C3—C2—H2	120.7	H13A—C13—H13C	109.5
O1—C3—C2	116.45 (19)	H13B—C13—H13C	109.5
O1—C3—C4	121.63 (18)	Si1—C14—H14A	109.5
C2—C3—C4	121.9 (2)	Si1—C14—H14B	109.5
C3—C4—C5	118.08 (18)	H14A—C14—H14B	109.5
C3—C4—C9	117.72 (19)	Si1—C14—H14C	109.5
C5—C4—C9	124.18 (19)	H14A—C14—H14C	109.5
C6—C5—C4	121.4 (2)	H14B—C14—H14C	109.5
C6—C5—H5	119.3	Si1—C15—H15A	109.5
C4—C5—H5	119.3	Si1—C15—H15B	109.5
C5—C6—C1	118.2 (2)	H15A—C15—H15B	109.5
C5—C6—C16	121.6 (2)	Si1—C15—H15C	109.5
C1—C6—C16	120.21 (19)	H15A—C15—H15C	109.5
O2—C7—O1	116.9 (2)	H15B—C15—H15C	109.5
O2—C7—C8	126.0 (2)	C6—C16—H16A	109.5
O1—C7—C8	117.04 (19)	C6—C16—H16B	109.5
C9—C8—C7	122.4 (2)	H16A—C16—H16B	109.5
C9—C8—H8	118.8	C6—C16—H16C	109.5
C7—C8—H8	118.8	H16A—C16—H16C	109.5
C8—C9—C4	119.28 (19)	H16B—C16—H16C	109.5
C8—C9—C10	123.50 (19)		
			
C11—N1—N2—N3	−0.5 (2)	C7—C8—C9—C4	−2.7 (3)
C10—N1—N2—N3	−179.99 (18)	C7—C8—C9—C10	177.1 (2)
N1—N2—N3—C12	0.4 (2)	C3—C4—C9—C8	2.3 (3)
C6—C1—C2—C3	0.1 (3)	C5—C4—C9—C8	−176.1 (2)
C7—O1—C3—C2	175.88 (19)	C3—C4—C9—C10	−177.55 (18)
C7—O1—C3—C4	−2.5 (3)	C5—C4—C9—C10	4.1 (3)
C1—C2—C3—O1	−178.51 (19)	C11—N1—C10—C9	100.6 (2)
C1—C2—C3—C4	−0.1 (3)	N2—N1—C10—C9	−79.9 (2)
O1—C3—C4—C5	178.70 (18)	C8—C9—C10—N1	8.1 (3)
C2—C3—C4—C5	0.4 (3)	C4—C9—C10—N1	−172.13 (18)
O1—C3—C4—C9	0.3 (3)	N2—N1—C11—C12	0.3 (2)
C2—C3—C4—C9	−178.0 (2)	C10—N1—C11—C12	179.78 (19)
C3—C4—C5—C6	−0.7 (3)	N1—C11—C12—N3	0.0 (2)
C9—C4—C5—C6	177.7 (2)	N1—C11—C12—Si1	176.29 (16)
C4—C5—C6—C1	0.6 (3)	N2—N3—C12—C11	−0.3 (2)
C4—C5—C6—C16	−178.9 (2)	N2—N3—C12—Si1	−176.77 (16)
C2—C1—C6—C5	−0.3 (3)	C13—Si1—C12—C11	157.8 (2)
C2—C1—C6—C16	179.2 (2)	C14—Si1—C12—C11	−83.7 (2)
C3—O1—C7—O2	−178.32 (19)	C15—Si1—C12—C11	35.6 (3)
C3—O1—C7—C8	2.1 (3)	C13—Si1—C12—N3	−26.5 (2)
O2—C7—C8—C9	−179.0 (2)	C14—Si1—C12—N3	92.0 (2)
O1—C7—C8—C9	0.5 (3)	C15—Si1—C12—N3	−148.7 (2)

### Hydrogen-bond geometry (Å, º)

**Table:**

*D*—H···*A*	*D*—H	H···*A*	*D*···*A*	*D*—H···*A*
C10—H10*A*···O2^i^	0.99	2.61	3.427 (1)	140
C11—H11···O2^i^	0.95	2.45	3.242 (1)	141

Symmetry code: (i) *x*, *y*+1, *z*.
