# Supplementary figures and images for: 6-Methyl-4-{[4-(tri­methyl­sil­yl)-1H-1,2,3-triazol-1-yl]meth­yl}-2H-chromen-2-one
Source: IUCrdata. 2020 Apr 3;5(Pt 4):x200427. doi: 10.1107/S2414314620004277 (PMC9462210; doi:10.1107/S2414314620004277)

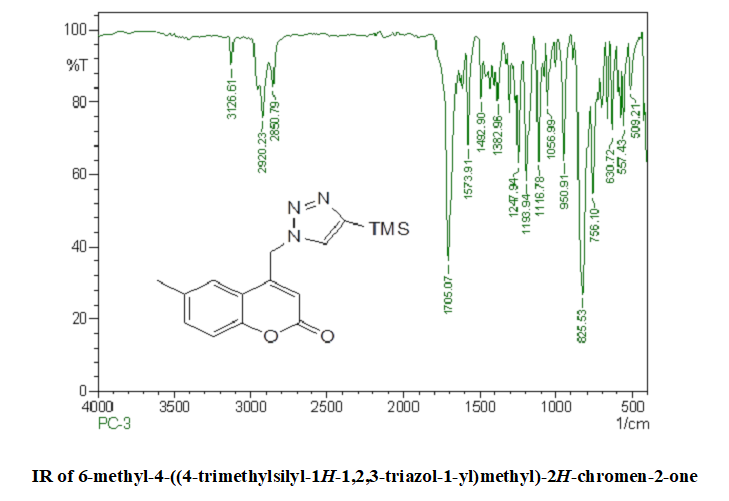

Supplement: Supplementary file 3 [file x-05-x200427-sup3.tif]

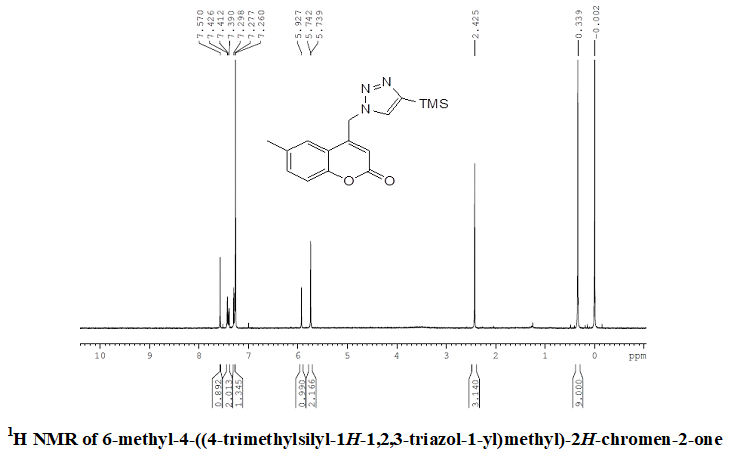

Supplement: Supplementary file 4 [file x-05-x200427-sup4.tif]

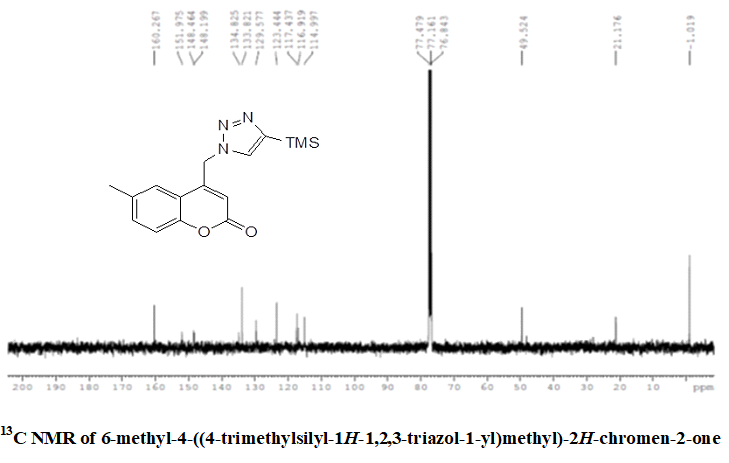

Supplement: Supplementary file 5 [file x-05-x200427-sup5.tif]

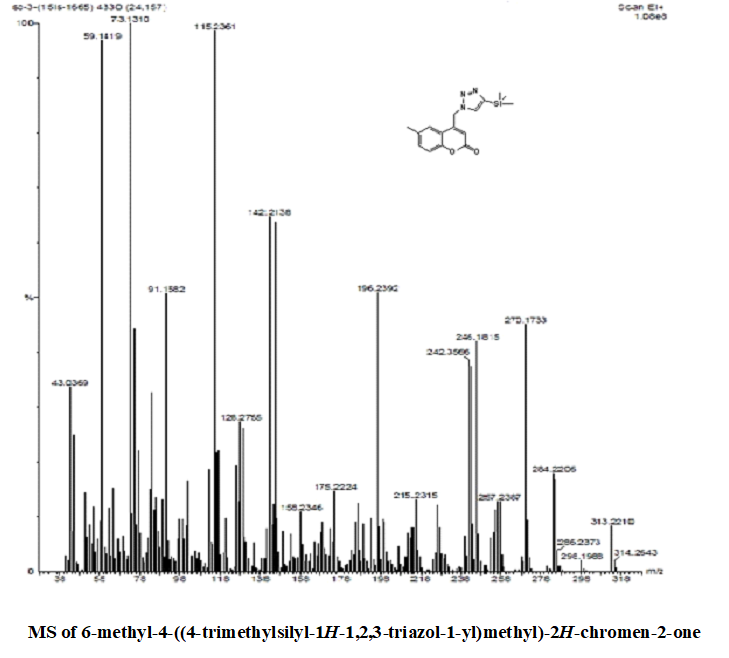

Supplement: Supplementary file 6 [file x-05-x200427-sup6.tif]
